# Retention in Care among HIV-Infected Pregnant Women in Haiti with PMTCT Option B

**DOI:** 10.1155/2016/6284290

**Published:** 2016-08-29

**Authors:** Jodie Dionne-Odom, Courtney Massaro, Kristen M. Jogerst, Zhongze Li, Marie-Marcelle Deschamps, Cleonas Junior Destine, Redouin Senecharles, Moleine Moles Aristhene, Joseph Yves Domercant, Vanessa Rouzier, Peter F. Wright

**Affiliations:** ^1^University of Alabama at Birmingham, Birmingham, AL, USA; ^2^Boston University School of Medicine, Boston, MA, USA; ^3^Geisel School of Medicine, Hanover, NH, USA; ^4^Biostatistics Shared Resource, Norris Cotton Cancer Center, Dartmouth College, Lebanon, NH, USA; ^5^Haitian Group for the Study of Kaposi's Sarcoma and Opportunistic Infections (GHESKIO), Port-au-Prince, Haiti; ^6^Immaculate Conception Hospital, Les Cayes, Haiti

## Abstract

*Background*. Preventing mother-to-child transmission of HIV relies on engagement in care during the prenatal, peripartum, and postpartum periods. Under PMTCT Option B, pregnant women with elevated CD4 counts are provided with antiretroviral prophylaxis until cessation of breastfeeding.* Methods*. Retrospective analysis of retention in care among HIV-infected pregnant women in Haiti was performed. Logistic regression was used to identify risk factors associated with loss to follow-up (LFU) defined as no medical visit for at least 6 months and Kaplan-Meier curves were created to show LFU timing.* Results*. Women in the cohort had 463 pregnancies between 2009 and 2012 with retention rates of 80% at delivery, 67% at one year, and 59% at 2 years. Among those who were LFU, the highest risk period was during pregnancy (60%) or shortly afterwards (24.4% by 12 months). Never starting on antiretroviral therapy (aRR 2.29, 95% CI 1.4–3.8) was associated with loss to follow-up.* Conclusions*. Loss to follow-up during and after pregnancy was common in HIV-infected women in Haiti under PMTCT Option B. Since sociodemographic factors and distance from home to facility did not predict LFU, future work should elicit and address barriers to retention at the initial prenatal care visit in all women. Better tracking systems to capture engagement in care in the wider network are needed.

## 1. Introduction

In Haiti, HIV prevalence decreased from 5.3% in 2000 to 2.7% in 2012 and the Haitian Ministry of Health (Ministère de la Santé Publique et de la Population or MSPP) has set a goal of eliminating vertical HIV transmission by the year 2018 [1]. Access to HIV testing and treatment during pregnancy has improved over the past decade with support from the US President's Emergency Plan for AIDS Relief (PEPFAR) and other funds to improve maternal and pediatric HIV care. Although early diagnosis and engagement in HIV care are critical to eliminating vertical transmission, postpartum loss to follow-up (LFU) is common and this is an important barrier to optimizing HIV outcomes in women and children [2–5]. The World Health Organization (WHO) Prevention of Mother-to-Child Transmission (PMTCT) “Option B” is a policy to provide prophylactic antiretroviral therapy (ART) for all pregnant women until cessation of breastfeeding for women who do not meet national treatment guidelines. Option B was the standard of care in pregnancy in Haiti during 2009–2012 and the threshold for long term ART after cessation of breastfeeding was a CD4 count <350 cells/mm^3^. In 2013, Haiti transitioned to PMTCT Option B+ which offers lifelong ART for all pregnant women with HIV.

The historical rate of vertical HIV transmission in Haiti has been documented by the Haitian Group for the Study of Kaposi's Sarcoma and Opportunistic Infections (GHESKIO): 27–37% in the pre-ART era, 10% in the early ART era (after 2003), and 1.9% in the current era [6–9]. Although studies have shown good ART adherence among adults with HIV in Haiti, pregnant women in the capital city of Port-au-Prince had a high LFU rate during 1999–2005 with 16% retention at 3 years [10–12]. LFU peaked in the immediate postpartum period and was significantly higher in women who did not meet CD4 criteria for ongoing ART. These findings have been replicated in Sub-Saharan Africa and in the United States [2–5, 13, 14]. With an average parity of 3.5, the PMTCT cascade and continuum of care in Haiti is relevant since many women are LFU during the postpartum period only to reengage in HIV care during a subsequent pregnancy. There are few studies of retention in care under PMTCT Option B.

In recognition of the “high risk” postpartum period for keeping women engaged in HIV care, changing ART recommendations, and the goal of eliminating mother-to-child transmission, we reviewed retention outcomes in HIV-infected pregnant women who received prenatal care from March 2009 to December 2012 at the departmental hospital facility in southern Haiti. PMTCT Option B was recommended during this entire period. The analysis was designed to understand the timing of LFU and sought to identify risk factors for LFU during pregnancy and after delivery. We also assessed whether retention in care improved between 2009 and 2012, coincident with earlier initiation of ART during pregnancy under Option B according to a change in national guidelines in June 2011.

## 2. Methods

### 2.1. Study Setting

Les Cayes is a city in Southern Haiti with a referral hospital (Immaculate Conception Hospital or HIC) that serves 700,000 people in the Department of the South. The hospital HIV team provides care for more than 3,000 patients, conducts HIV training, and performs monitoring and evaluation efforts to improve HIV outcomes with PEPFAR funding. These efforts are coordinated by GHESKIO with collaboration from MSPP and the Centers for Disease Control and Prevention (CDC) Haiti office. Funds provided through the GHESKIO PEPFAR program supported activities by a Dartmouth team designed to introduce critical analysis and innovation at the HIC site. At HIC, all women seen for prenatal care are offered an HIV test at the initial visit with follow-up screening 12 weeks later and facility delivery is strongly recommended. During the study period of 2009–2012, approximately 300 pregnant women were tested for HIV each month and 50 HIV-infected women were followed up monthly in the HIC PMTCT clinic staffed by midwives and nurses. Regional HIV seroprevalence among women of ages 15–49 was 2.4% in 2012 [15]. Pregnant women with a new HIV diagnosis were given a follow-up appointment to start ART within two weeks. Patients were scheduled for monthly follow-up visits during pregnancy and 1-2 postpartum visits before care was transferred back to the adult HIV service with quarterly follow-up visits for clinically stable patients. Personal information was kept up-to-date and women who missed PMTCT appointments were contacted by clinic staff by telephone. If women were unavailable by telephone, community health workers were sent to look for them based on their home address.

**Figure 1:**
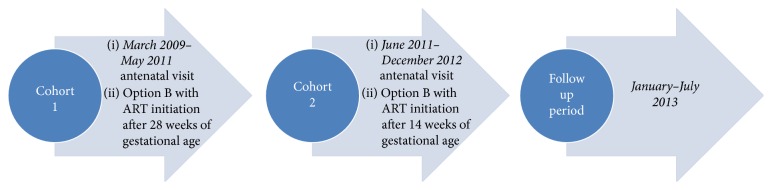
Study timeline for HIV-infected pregnant women.

Recommendations about ART timing during pregnancy under PMTCT Option B in Haiti changed in June 2011 (Figure 1) [16]. During March 2009–May 2011, the standard regimen of twice daily zidovudine, lamivudine, and nevirapine was initiated after 28 weeks of gestation, irrespective of CD4 count. Women who were already on ART when they became pregnant were continued on their regimen; after delivery, breastfeeding and ongoing ART were recommended. According to the WHO PMTCT Option B, women who did not meet contemporaneous treatment guidelines (CD4 <350 cells/mm^3^) were advised to stop ART one week after breastfeeding cessation. After June 2011, ART initiation was recommended after 14 weeks of gestation for women who were not already taking HIV medications. Periodicity of follow-up care was the same for women in PMTCT and adult HIV clinics, irrespective of CD4 count. Women initiating lifelong ART therapy during pregnancy under Option B+ were not included in this study.

### 2.2. Study Population

HIC hospital and clinic records from March 2009 until November 2012 were reviewed to identify all HIV-infected pregnant women who had been seen in prenatal clinic at least once. Pregnancy dating was based on the patient's report of the last menstrual period (LMP). Data was collected from paper records and an electronic medical record system (iSanté). Follow-up retention data was collected through July 2013 (Figure 1).

### 2.3. Data Collection

Deidentified retrospective data was collected for analysis and the study was approved by the HIC Hospital Director in lieu of an established IRB in Les Cayes and by the Dartmouth College Committee for the Protection of Human Subjects with a waiver of informed consent. The initial CD4 count at time of HIV diagnosis and the CD4 cell count closest to the date of delivery were captured and the timing of HIV diagnosis and ART initiation, the distance (in kilometers) from home to the HIC hospital, relationship status, delivery date, and delivery location (home or facility) were entered into a secure, anonymous data set. For women who were LFU, chart notes were reviewed to see whether the reason for LFU was documented based on information provided by the social workers or community health workers. Reasons for LFU included transfer of care to an alternate facility, moving away, disinterest in follow-up, insufficient financial resources, stigma, or death. In an attempt to further track women who were LFU, we worked with the National Alliance of State and Territorial AIDS Directors (NASTAD), a nongovernmental organization that maintained a national HIV case surveillance system on behalf of the Haitian Ministry of Health. This database retrospectively identified HIV-infected women who presented for follow-up care in other facilities but it was not designed to provide information to care teams in real time [17].

### 2.4. Definitions

The study cohort was divided into 2 groups: women who were actively in care (defined as a medical visit at least every six months until the study ended in July 2013) and those who were LFU (no medical visit for a period greater than six months during or after pregnancy) (Figure 1). There is no standardized definition for retention in HIV care, but the 6-month gap measure is consistent with other published studies in the postpartum setting [14, 18, 19]. Demographic data on women with more than one birth was reported at the time of each pregnancy but multiple events analysis was not performed (Table 1). Women were also separated into an earlier and a later cohort based on an ART initiation date that fell before or after June 1, 2011; the date that corresponded to a change in national ART guidelines recommending initiation after 14 weeks of gestation instead of 28 weeks (see Figure 1). For women who were not initiated on ART, the date of the initial visit in antenatal care (ANC) clinic was used for categorization.

### 2.5. Statistical Methods

Frequency and percent for categorical variables and means and standard deviations for continuous variables were calculated. Chi-square test for categorical variables and two-sample *t*-test for continuous variables were used for comparison of subjects actively in care and LFU. Significance was set at 5% and *p* values were two-sided. Considering that LFU was not rare, we used a univariate and multivariate log-binomial model (i.e., log link function) in logistic regression, to estimate relative risk (RR) and confidence intervals for all possible factors separately. A subset analysis was performed for women who had at least 2 antenatal care visits during the study period. Observations with missing values were not included in the analysis and imputation techniques for missing data were not used. Kaplan-Meier (KM) survival analysis was used to estimate the probability of remaining in care during and after pregnancy for the entire cohort and the dichotomized cohort based on the date of ART initiation or entry to ANC care.

## 3. Results

There were 438 HIV-infected pregnant women in this study who received prenatal care at HIC in Southern Haiti between March 2009 and December 2012 (Table 1). Two hundred and seven women in the retained group had 225 births (14 women had 2 births and 2 women had 3 births) and 231 women in the LFU cohort had 238 births (7 women had 2 births). The mean age at the time of enrollment in prenatal care was 28 years. Younger women (<25 years old) were more likely to be in the LFU group (*p* = 0.02) (Table 1). Most women (75.2%) with available CD4 cell counts had testing within 6 months of their delivery date but one in four women had a CD4 value collected within 6–12 months of delivery. The average CD4 count among women was 581 cells/mm^3^, but the range was broad (28–1694 cells/mm^3^). There was no significant difference in the mean CD4 closest to delivery between the women who remained actively in care and those who were LFU. There was also no difference in the likelihood of an initial CD4 <350 in terms of retention in care (to distinguish between women who were eligible for long term ART versus short term prophylaxis under Option B guidelines). Most women who were retained in care had been diagnosed with HIV prior to pregnancy (134/216 or 62%), while women in the LFU group were more likely to have had HIV diagnosed during pregnancy (124/212 or 58.5%), *p* < 0.0001. In addition, there were more women with ART initiation before pregnancy in the active group (29.8%) compared to the LFU group (10.9%). A majority of women were married or cohabitating (70%) and facility delivery rates were approximately 75% although many women did not have the location of birth documented in their medical record.

**Figure 2:**
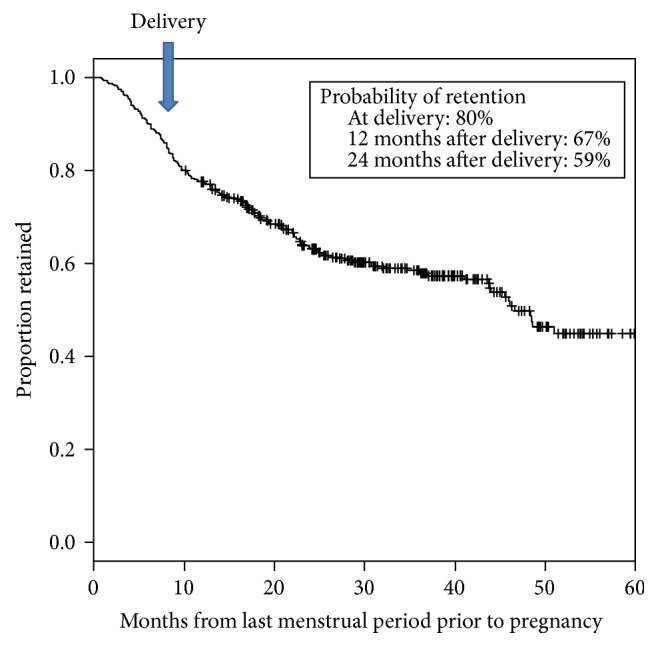
Retention in care during and after pregnancy for all women.

**Figure 3:**
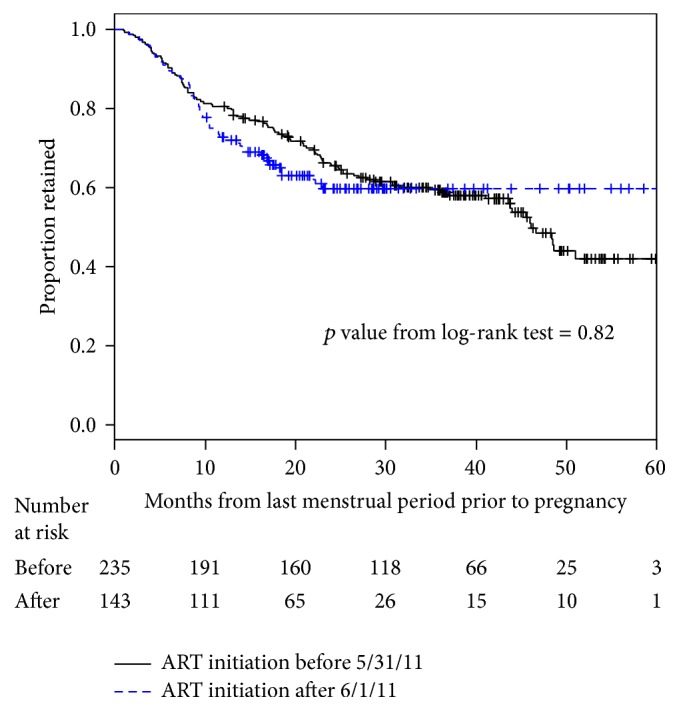
Retention in care by cohort number based on ART initiation date.

Overall, only 80% of pregnant women were retained in care at delivery (Figure 2). After delivery, retention rates fell to 67% at 12 months and to 59% by 24 months. Retention is shown with a Kaplan-Meier curve that has a steady decline and a suggestion of a plateau after 3 years. Due to the study design, the length of follow-up time varied and women who delivered after August 2011 had less than 24 months of follow-up time. This is shown with the number “at risk” shown on the KM curve in Figure 3. Many of the women who were LFU had their last facility visit during pregnancy (60%) and many women were only seen once at the facility (105/231 or 45% of the LFU cohort) (Table 1). Women with a single visit were more likely to have their initial HIV test and ART initiation during the 3rd trimester of pregnancy (*p* < 0.001) but age, CD4 count, relationship status, delivery location, and distance from home were not significantly different compared to women who were LFU after multiple clinic visits (data not shown). Following the change in guidelines in June 2011, ART was initiated earlier in pregnancy (Table 2). In the earlier cohort, many women were not started on ART until the 3rd trimester (29.5%) but, in the later cohort, most women were started on ART during the 2nd trimester (33.1%). Similar numbers (20.8% and 18.3%) in both groups were not started on ART at all. When the retention rates were stratified by cohort, there was no improvement in retention seen in 2011-2012 compared to 2009–2011, despite the earlier ART initiation (*p* = 0.82, Figure 3).

Results from the univariate and multivariate analyses are shown in Table 3(a). In the unadjusted model, HIV testing during pregnancy compared to before pregnancy was highly associated with LFU (RR 1.5, 95% CI 1.3–1.9). LFU was also associated with later (3rd trimester) ART initiation (RR 2.1, 95% CI 1.5–3) with a trend toward significance for 2nd trimester ART initiation (RR 1.47, 95% CI 0.99–2.2). Older women (>35 years) also had a trend toward lower LFU rates compared to women who were 25–34 years old (RR 0.76, 95% CI 0.57–1.02) in the unadjusted model. In the multivariate model, the only factor associated with LFU was never having started on ART (aRR 2.29, 95% CI 1.4–3.8). In the subgroup analysis of women who attended at least 2 ANC visits, the association between LFU and never starting ART persisted (aRR 2.11, 95% CI 1.3–3.4) (Table 3(b)). Reasons for LFU in this group were only available for a subset of women (46 or 20% of the LFU group) and the two most common reasons cited were that women had moved (37%) or transferred care to another site (32.6%) (Table 4). Cohort data from the HIV case surveillance system maintained by NASTAD was collected in the search for additional follow-up information on the LFU cohort but very few (16/231 or 6.9%) were captured in their system as having presented for care elsewhere. It was not possible to determine whether or not these women were actively engaged in care.

## 4. Discussion

This study documents some of the challenges in retaining pregnant and postpartum women in care and the limitations of ART provided under PMTCT Option B. Twenty percent of pregnant women enrolled in prenatal care at one large facility in Haiti were no longer in care at the time of delivery and retention in care (defined as a 6-month gap without a medical visit) was 67% at 1 year and 59% by 2 years after delivery. These high LFU rates occur in a setting with many beneficial attributes; a population in close proximity to a departmental hospital, favorable ART access, and a capable care team that is integrated with community health outreach efforts. The LFU rates in this study are similar to those documented in other settings. In one study of 300 pregnant women in South Africa with newly diagnosed infection, only 40% were retained in care through 6 months postpartum and retention was lowest among women who were not ART eligible [13]. Pregnancy itself has been shown to be associated with poor retention in care in several countries [3, 14, 20, 21]. One study in Malawi with early rollout of universal ART under Option B+ documented improved retention at 6 months postpartum (83%) although additional, larger retention studies in the era of Option B+ are ongoing [22–24]. The single factor associated with LFU in our multivariate analysis was never starting ART. This association between ART initiation and retention was noted for the entire cohort and the subset with multiple ANC visits. Other studies have shown a similar benefit to ART initiation and the integration of ART services with antenatal care on retention in care in pregnancy [25–28].

We were unable to identify other predictors of retention in pregnant women with HIV in Haiti. A diagnosis of HIV during pregnancy and later ART initiation during the 3rd trimester were associated with LFU in the univariate analysis and this supports the need for prepregnancy HIV diagnosis and engagement in care. However, in our model, these and other factors including age and distance from home to facility were not associated with LFU after adjustment. We did not capture information about maternal educational levels or wealth status although these are potential predictor of retention. We did explore whether or not the initial CD4 count predicted retention in care (with the hypothesis that women with higher CD4 counts advised to receive short term ART are at risk for LFU) but this factor was nonsignificant in our model. Retention in HIV care during and after pregnancy is complex and likely multifactorial. Other studies of HIV-infected pregnant women in Tanzania have shown the importance of age as younger women (<24 years) were more likely to refuse ART prophylaxis [29]. Although we were surprised by the lack of association between LFU and the distance from home to clinic, 2 of 3 women in this study lived in the immediate catchment area with residence within 5 kilometers of the facility. The HIC facility may not be serving optimally as a referral center for HIV care during pregnancy throughout the department and this data suggests that additional outreach is needed to reach pregnant women with HIV-infection who reside farther away from HIC. Louis et al. examined factors associated with the timing of presentation for general HIV care in central Haiti and identified that living a distance >2 hours from a facility was associated with late presentation. They also identified that socioeconomic factors (lack of latrine) were associated with LFU [30]. In other studies in Africa and the US, barriers to postpartum retention included distance, lack of money, perceived poor treatment at the clinic, stigma, limited social support, and fear of disclosure [31–33].

Excellent retention at every step of the PMTCT cascade is necessary to optimize outcomes, as documented by the PEARL study in Africa and other modeling studies [34–36]. Dropout from the care cascade among pregnant women may seem counterintuitive, since pregnancy is generally associated with increased engagement with healthcare services. However, this temporary increase in engagement often does not extend into the postpartum period, particularly for women with structural barriers to care (such as transportation) and new responsibilities after a birth. There is also the critical issue of stigma which can be heightened during pregnancy, can be difficult to measure, and was not assessed in this study. It is interesting that the prenatal HIV team at HIC noted that many of the women with a single facility visit cited concerns around HIV status disclosure. Other women requested repeated HIV testing, stating that they did not believe the previous positive results. Following successful linkage to care, tracking individuals who are subsequently LFU presents many challenges. In Haiti, community health workers travel long distances and put significant effort into keeping women engaged in HIV care during and after pregnancy. Careful search for these “lost” individuals with the provision of additional time and resources is helpful in determining true retention rates. In some studies, up to 50% of the lost cohort can be tracked down, but real-time tracking is challenging for any team on the ground. A better system of HIV surveillance is needed to track increasingly mobile patients as they present for care at various facilities [37–39]. This has particular relevance in the postpartum setting since women must transition from attending ANC clinic to adult HIV clinic in addition to scheduling pediatric and immunization visits for the infant.

Several community based strategies have shown promise in increasing rates of PMTCT retention in care [40]. In Haiti, we introduced community based HIV care for adults with “Groups of Six” and HIC has support and education groups called “Clubs des Meres” for prenatal care [41]. Both groups could be easily adapted to the postpartum setting. GHESKIO is exploring same day ART initiation on the day of HIV diagnosis as a potential way to decrease barriers to care [26, 42] (personal communication, S. Koenig). One success shown in the current analysis is high facility delivery rates among HIV-infected women (78.5% and 71.3% in the active and lost to follow-up groups, although many women did not have the birth location documented). For comparison, the facility delivery rate among HIV-uninfected women in 2012 was 36% [15]. This improvement may be explained by frequent reminders from the team during antenatal care about the importance of facility delivery for women with HIV.

This study has several limitations. Data is incomplete for the women who were LFU and information about their outcomes was limited. This increases the risk of bias and there may have been an association between LFU and variables such as CD4 count, distance from home to facility, or relationship status that was not detected due to missing data. Also, women who received antenatal care between August and December of 2012 had a shorter follow-up period (<12 months) given outcome ascertainment in July 2013. Many women in the cohort had only one visit with the antenatal care team which may limit generalizability although this is an important finding to document and women with more than one visit had similar characteristics compared to the rest of the group with LFU. These are results from a large departmental hospital setting which may be not be generalizable to retention in care in more rural areas. Fortunately, this is a more stable population compared to other areas of Haiti since it was less affected by the earthquake that took place in Léogâne in January 2010 but some movement in and out of the area did occur. The study strengths include the duration of follow-up for many participants, the inclusion of data captured by community health worker efforts, and the documentation of retention under Option B to provide comparison data for countries collecting retention outcomes in the setting of PMTCT Option B+.

## 5. Next Steps

Poor retention in care in the postpartum setting is a common and important problem worldwide. Care engagement should be supported from the initial ANC visit and ART initiation should be prioritized. Future studies should assess structural, community, facility, and individual level barriers to care in order to guide tailored interventions. The development of a robust computer system to track women with HIV receiving care at various facilities would be a major advance toward documenting actual postpartum retention rates. In 2013, the Haitian Ministry of Health (MSPP) implemented an enhanced perinatal HIV surveillance tracking system called SAFE (Surveillance Active de la Femme Enceinte Seropositive) to track women during pre- and postpartum care with timed reminders sent to case managers for key events. Reporting is not yet universal but implementation and training are ongoing. Retention outcomes in pregnancy in Haiti should be measured in light of this improved surveillance system.

## Figures and Tables

**Table 1:** Participant characteristics^*∗∗*^.

	Active in care *n* = 225 births *N* (%)	Lost to follow-up *n* = 238 births *N* (%)	*p* value
Age (years)	*n* = 225	*n*=233^*∗∗*^	
Mean ± SD (range)	29.1 ± 6.3	27.5 ± 6.2	0.006
Range	16–44	14–46	
<25	59 (26.2)	82 (35.2)	0.02
25–34	111 (49.3)	116 (49.8)	
35+	55 (24.4)	35 (15)	

Timing of initial HIV test	*n* = 216	*n* = 212	
Before pregnancy	134 (62)	88 (41.5)	<0.0001
During pregnancy	82 (38)	124 (58.5)	

Initial CD4	*n* = 224	*n* = 140	0.70
≤350 cells/mm^3^	78 (34.8)	46 (32.9)	
>350 cells/mm^3^	146 (65.2)	94 (67.1)	

Mean CD4 near delivery	*n* = 217	*n* = 126	
Mean ± SD	593 ± 273	559 ± 302	0.29
Range	30–1471	28–1694	
<200	6 (2.8)	12 (9.5)	0.02
200–349	32 (14.8)	19 (15.1)	
350+	179 (82.5)	95 (75.4)	

Timing of ART initiation	*n* = 225	*n* = 238	
Before pregnancy	67 (29.8)	26 (10.9)	<0.0001
1st trimester	12 (5.3)	6 (2.5)	
2nd trimester	60 (26.7)	42 (17.6)	
3rd trimester	51 (22.7)	74 (31.1)	
Postpartum	25 (11.1)	8 (3.4)	
Never started	10 (4.4)	82 (34.5)	

Relationship status	*n* = 224	*n* = 183	
Married/cohabitating	156 (69.6)	129 (70.5)	0.67
Widowed	5 (2.2)	2 (1.1)	
Separated	12 (5.4)	7 (3.8)	
Single	30 (13.4)	22 (12)	
Unknown	21 (9.4)	23 (12.6)	

Delivery location	*n* = 158	*n* = 115	
Facility	124 (78.5)	82 (71.3)	0.17
Home	34 (21.5)	33 (28.7)	

Distance home to hospital	*n* = 224	*n* = 184	
Mean ± SD	9.6 ± 16.7	8.4 ± 12.6	0.39
Range	0–101	0–78	
0–5 km	149 (66.5)	124 (67.4)	0.56
5–20 km	33 (14.7)	21 (11.4)	
>20 km	42 (18.8)	39 (21.2)	

Cohort timing^*∗*^			
Cohort 1: March 2009–May 2011	131 (58.2)	157 (66)	0.09
Cohort 2: June 2011–November 2012	94 (41.8)	81 (34)	

Number of antenatal care visits			
1	0 (0)	105 (44.1)	<0.0001
2 or more	225 (100)	133 (65.9)	

Timing of LFU		*n* = 238	
During pregnancy	N/A	143 (60)	NA
0–3 months postpartum		21 (8.8)	
4–6 months postpartum		13 (5.5)	
7–9 months postpartum		16 (6.7)	
10–12 months postpartum		8 (3.4)	
13–18 months postpartum		18 (7.6)	
19–24 months postpartum		6 (2.5)	
>24 months postpartum		13 (5.5)	

^*∗*^Based on ART initiation date (or entry to care if ART was not started). Per MSPP guidelines, ART was started at 28 weeks, 2009–2011. In June 2011, women were started on ART at 14 weeks.

^*∗∗*^ *n* shows available data for each variable excluding missing data. Observations with missing values were not included in the analysis.

**Table 2:** Timing of ART initiation based on change in guidelines in June 2011^*∗*^.

	Cohort 1 (*n* = 288) *n* (%)	Cohort 2 (*n* = 175) *n* (%)	Total (*n* = 463) *n* (%)
Before pregnancy	84 (29.2)	9 (5.1)	93 (20)
1st trimester	6 (2.1)	12 (6.9)	18 (3.9)
2nd trimester	44 (15.3)	58 (33.1)	102 (22)
3rd trimester	85 (29.5)	40 (22.9)	125 (27)
After pregnancy	9 (3.1)	24 (13.7)	33 (7.1)
Never started	60 (20.8)	32 (18.3)	92 (20)

^*∗*^Per MSPP guidelines, ART was started at 28 weeks in 2009–2011. In June 2011, women were started on ART at 14 weeks.

**(a):** 

Variable	Unadjusted		Adjusted	
	Relative risk (95% CI)	*p* value	Relative risk (95% CI)	*p* value
Age				
<25	1.14 (0.94, 1.38)	0.18	1.18 (0.89, 1.58)	0.25
25–34	Reference	NA	Reference	NA
35+	0.76 (0.57, 1.02)	0.06	0.78 (0.52, 1.19)	0.25

HIV test timing				
Before pregnancy	Reference	NA	Reference	NA
During pregnancy	1.52 (1.25, 1.85)	<0.0001	1.17 (0.87, 1.58)	0.29

Initial CD4				
≤350	Reference	NA	Reference	NA
>350	1.06 (0.8–1.39)	0.7	0.81 (0.58–1.12)	0.21

ART start timing				
Before pregnancy	Reference	NA	Reference	NA
1st trimester (2–14 wks)	1.19 (0.57, 2.47)	0.64	1.03 (0.49, 2.17)	0.95
2nd trimester (15–28 wks)	1.47 (0.99, 2.2)	0.06	1.19 (0.73, 1.96)	0.49
3rd trimester (29–42)	2.12 (1.48, 3.03)	<0.0001	1.53 (0.96, 2.45)	0.07
Postpartum	0.87 (0.44, 1.72)	0.68	0.82 (0.38, 1.75)	0.6
Never started	3.19 (2.28, 4.5)	<0.0001	2.29 (1.39, 3.78)	<0.01

Relationship				
Married/cohabitating	Reference	NA	Reference	NA
Widowed	0.63 (0.19, 2.05)	0.44	1.14 (0.37, 3.56)	0.82
Separated	0.81 (0.45, 1.49)	0.5	0.8 (0.36, 1.78)	0.58
Single	0.93 (0.66, 1.32)	0.7	0.95 (0.66, 1.37)	0.78
Unknown	1.15 (0.85, 1.57)	0.36	1.32 (0.91, 1.92)	0.15

Distance from home to hospital (KM)	1 (0.99, 1)	0.44	1 (0.99, 1.01)	0.51

Cohort timing				
Cohort 1: March 2009–May 2011	1.18 (0.97, 1.43)	0.09	1 (0.75, 1.33)	0.99
Cohort 2: June 2011–November 2012	Reference	NA	Reference	NA

^*∗*^Using a logistic model for binomial outcome with log link function to estimate relative risk.

**(b):** 

Variable	Unadjusted		Adjusted	
	Relative risk (95% CI)	*p* value	Relative risk (95% CI)	*p* value
Age				
<25	1.19 (0.89, 1.58)	0.24	1.18 (0.89, 1.57)	0.26
25–34	Reference	NA	Reference	NA
35+	0.69 (0.45, 1.06)	0.09	0.83 (0.57, 1.2)	0.32

HIV test timing				
Before pregnancy	Reference	NA	Reference	NA
During pregnancy	1.32 (1.01, 1.72)	0.05	1.09 (0.82, 1.45)	0.54

ART start timing				
Before pregnancy	Reference	NA	Reference	NA
1st trimester (2–14 wks)	1.3 (0.62, 2.74)	0.48	1.08 (0.55, 2.11)	0.83
2nd trimester (15–28 wks)	1.28 (0.8, 2.02)	0.30	1.14 (0.72, 1.82)	0.57
3rd trimester (29–42)	1.81 (1.2, 2.74)	0.00	1.47 (0.95, 2.27)	0.08
Postpartum	0.95 (0.47, 1.91)	0.88	0.9 (0.45, 1.78)	0.76
Never started	2.73 (1.8, 4.14)	<0.0001	2.11 (1.31, 3.39)	<0.01

Relationship				
Married/cohabitating	Reference	NA	Reference	NA
Widowed	0.78 (0.24, 2.53)	0.67	1.13 (0.41, 3.11)	0.81
Separated	0.8 (0.38, 1.7)	0.56	0.85 (0.42, 1.71)	0.65
Single	0.9 (0.58, 1.41)	0.66	0.96 (0.67, 1.37)	0.81
Unknown	1.25 (0.86, 1.83)	0.24	1.3 (0.9, 1.89)	0.16

Distance from home to hospital (KM)	1 (0.99, 1.01)	0.72	1 (0.99, 1.01)	0.68

Cohort timing (based on ART initiation)				
Cohort 1: before 5/31/2011	1.09 (0.83, 1.45)	0.53	1 (0.76, 1.31)	0.98
Cohort 2: after 6/1/2011	Reference	NA	Reference	NA

Initial CD4				
≤350	Reference	NA	Reference	NA
>350	0.99 (0.74, 1.33)	0.94	0.81 (0.6, 1.11)	0.19

^*∗*^Using a logistic model for binomial outcome with log link function to estimate relative risk.

**Table 4:** Reasons for loss to follow-up documented by community health workers (*n* = 46).

Reason for LFU	Number (%)
Moved away	17 (37)
Transferred care to another site	15 (32.6)
Did not accept their diagnosis	5 (10.9)
Did not want others to find out	4 (8.7)
Died	3 (6.5)
Too ill to leave home	2 (4.3)
